# Genome-wide assessment of runs of homozygosity and inbreeding in Inner Mongolia cashmere goats reveals candidate genes for economic traits

**DOI:** 10.3389/fvets.2026.1796462

**Published:** 2026-03-12

**Authors:** Yunpeng Qi, Youjun Rong, Mingzhu Zhang, Xingle Wang, Xiaofang Ao, Fangzheng Shang, Yanjun Zhang, Ruijun Wang

**Affiliations:** 1 College of Animal Science, Inner Mongolia Agricultural University, Hohhot, China; 2 Key Laboratory of Mutton Sheep Genetics and Breeding, Ministry of Agriculture, Hohhot, China; 3 Northern Agriculture and Livestock Husbandry Technology Innovation Center, Chinese Academy of Agricultural Sciences, Hohhot, China

**Keywords:** candidate genes, inbreeding coefficients, Inner Mongolia cashmere goats, ROH islands, runs of homozygosity

## Abstract

**Introduction:**

To facilitate the conservation and sustainable utilization of the Inner Mongolia Cashmere Goat (IMCG)-an important genetic resource and economically valuable breed-this study performed a genome homozygosity assessment of its Erlangshan subtype population, analyzed the distribution characteristics of runs of homozygosity (ROH), and compared inbreeding coefficients estimated using different methods.

**Methods:**

A total of 384 individuals were genotyped using the Illumina Goat SNP70K BeadChip. PLINK software was employed to conduct ROH analysis, estimate genomic inbreeding coefficients, and identify ROH islands based on the obtained SNP data.

**Results:**

The results revealed 3,622 ROH segments in the population, with an average total ROH length of 71.48 Mb per individual. The inbreeding coefficients based on ROH (F_ROH_) and observed homozygosity (F_HOM_) were 0.0290 and −0.0126, respectively, showing a strong positive correlation (*r* = 0.97). In addition, 14 ROH islands were identified on chromosomes 2, 5, 7, 8, 9, 13, 17, 19, 24, 25, and 26. Within these regions, 238 genes were annotated, including key genes such as *HDAC1*, *MIR21*, *DNASE1L2*, and *ACACA*, which may be implicated in reproductive traits, hair follicle development, and milk fat metabolism.

**Conclusion:**

This study systematically characterized ROH patterns in IMCG, indicating a generally low level of genomic inbreeding in the population, though certain individuals still require attention. The identification of genes related to economically important traits further reveals the genetic distinctiveness and breeding potential of IMCG, providing valuable insights for informing future conservation and breeding strategies.

## 1 Introduction

China is rich in indigenous genetic resources of cashmere goats. Among them, the Inner Mongolia Cashmere Goat (IMCG) represents a distinctive breed consisting of three subtypes: Albas, Erlangshan, and Alashan (1). Known for producing high-quality meat and fine cashmere, IMCG is a vital component of China’s cashmere goat genetic resources (2). To date, research has primarily focused on selective breeding for cashmere traits, differential gene expression, and molecular regulation of hair follicle development, while analyses of runs of homozygosity (ROH) patterns remain limited (1). ROH analysis based on genomic data can reveal the extent of inbreeding, recent population bottlenecks, and signatures of directional selection. Therefore, this study addresses this knowledge gap by systematically describing the occurrence and distribution of ROH in the IMCG population using medium-density single nucleotide polymorphisms (SNPs) chip data, thereby laying a foundation for subsequent genetic studies.

In genomic research, ROH refers to continuous homozygous DNA segments in diploid organisms that are inherited identically by descent from common ancestors (3). Because genetic recombination breaks such homozygous fragments over generations, ROH length serves as an important indicator for tracing the timing of inbreeding events: long ROH segments typically reflect recent inbreeding, whereas shorter ones indicate more ancient common ancestry (4). With the marked reduction in genotyping costs and the rapid increase in genomic data, goat breeding has entered the era of genomic selection. Consequently, methods for estimating inbreeding coefficients are gradually shifting from traditional pedigree-based approaches to genomic computations based on ROH (5). Compared with indirect pedigree-based estimates, the genomic inbreeding coefficient derived from ROH (F_ROH_) offers clear advantages, especially when pedigree records are incomplete or missing.

ROH analysis also facilitates the identification of genomic regions with high frequencies of homozygosity, termed ROH islands. Detailed examination of these regions can provide deeper insights into genomic intervals under selective pressure, thereby revealing key information about population genetic adaptation and evolutionary history (6). The detection of ROH islands not only complements genome-wide association studies but also helps uncover species-specific important genes. In recent years, genome-wide analyses of ROH have been extensively applied across diverse livestock and poultry species to characterize population structure, estimate genomic inbreeding, and detect signatures of selection. For instance, Ma et al. (7) employed a genome-wide ROH approach in ovine populations, revealing breed-specific inbreeding patterns and detecting candidate genes associated with wool quality and reproductive efficiency. Hervás-Rivero et al. (8) through the mapping of ROH islands in autochthonous Spanish beef cattle breeds, uncovered genes related to muscle growth (e.g., *MSTN*) and fertility under shared ancestral selection pressures. Rostamzadeh Mahdabi et al. (9) via comparative ROH island analyses in indigenous and commercial chickens, precisely pinpointed candidate loci associated with disease resistance, heat tolerance, and reproduction. These cross-species applications demonstrate the powerful role of ROH analysis in elucidating the genetic basis of economically important traits and guiding breeding strategies. Therefore, this study aims to systematically identify and characterize ROH patterns in the IMCG (Erlangshan subtype) population using medium-density SNP chip data. We assess its inbreeding status, screen potential candidate genes within ROH islands associated with key production traits, and provide foundational genomic information to support future conservation and breeding programs.

## 2 Materials and methods

### 2.1 Experimental animals and its care

All animal experiments were performed by the Guidelines for Experimental Animals of the Ministry of Science and Technology (Beijing, China) and were approved by the Scientific Research and Academic Ethics Committee of Inner Mongolia Agricultural University and the Biomedical Research Ethics of Inner Mongolia Agricultural University (Approval No. [2020] 056).

### 2.2 Animal origin and DNA extraction

A total of 384 IMCG (Erlangshan subtype) individuals (104 rams and 280 ewes) were sampled from the Erlangshan Pasture (108°31′E, 41°26′N, altitude 1,248 m) of Inner Mongolia Beiping Textile Co., Ltd. After ear tissue collection from all individuals, genomic DNA was extracted using the standard phenol-chloroform method (10). DNA purity (260/280 nm ratio) was assessed with a Nanodrop 2000 spectrophotometer (Thermo Fisher Scientific Inc., United States). Qualified DNA samples were stored at −80 °C for subsequent genotyping.

### 2.3 Genotyped and quality control

Genotyping of qualified samples was performed using the Illumina Goat SNP70K BeadChip (Inner Mongolia Agricultural University, China). Genotype quality control was conducted with PLINK (V1.90) software (11). Single nucleotide polymorphisms (SNPs) were removed if they met any of the following criteria: (i) call rate < 90%; (ii) minor allele frequency < 0.05; (iii) Hardy–Weinberg equilibrium *p*-value < 1 × 10^−6^; or (iv) individual genotype missing rate >10%. Additionally, SNPs located on sex chromosomes and those with ambiguous chromosomal positions were also excluded. After quality control, 50,933 SNPs from 380 animals were retained for subsequent analyses. The SNPs dataset has been deposited in the Figshare database.^1^

### 2.4 Runs of homozygosity detection and classification

PLINK (V1.9) software (11) employs a sliding window algorithm to analyze the genome and identify ROH. In consideration of the characteristics of the medium-density Illumina Goat SNP70K BeadChip, the following parameter set was applied in this study: (1) a sliding window of 50 SNPs across the genome; (2) to minimize the number of false positive ROH, the minimum number of SNPs constituting the ROH was calculated using the method proposed by Lencz et al. (12):


$$l=\frac{\log_{e}\alpha /n_{s}\times n_{i}}{\log_{e}(1-\mathrm{het})}$$

where α is the percentage of false positive ROH (set to 0.05 in this study), $n_{s}$ is the number of SNPs per individual, $n_{i}$ is the number of individuals, het is the heterozygosity across all SNPs; (3) up to one missing SNP and one heterozygous genotype were allowed per ROH to accommodate potential genotyping errors (13); (4) a maximum gap between consecutive SNPs of 1 Mb and minimum SNP density was set to 1 SNP per 100 kb; (5) to distinguish true autozygous segments from short homozygous tracts likely arising from linkage disequilibrium (LD), we opted to set a minimum length threshold of 1 Mb for ROHs instead of performing LD pruning (14). The parameter set chosen here represents a balanced strategy widely used in livestock genomics research for medium-density SNP array data.

### 2.5 Runs of homozygosity distribution and inbreeding coefficients

First, the mean number of ROH (MN_ROH_), mean length of ROH (ML_ROH_), and the total number of ROH was estimated for each animal. The number of ROH on different chromosomes and the percentage of chromosomes covered by ROH were also calculated. This coverage percentage for each chromosome was calculated by dividing the total length of ROH on that chromosome by the full length of the corresponding chromosome as per the ARS1.2 goat reference genome assembly. Next, the lengths of all ROH were categorized into four categories: 1–5 Mb, 5–10 Mb, 10–20 Mb, and >20 Mb, and the number of ROH in each category on each chromosome was counted. In addition, we estimated and analyzed two measures of inbreeding: the coefficient based on observed homozygosity (F_HOM_) and that based on ROH (F_ROH_). F_ROH_ was estimated for each individual using PLINK according to McQuillan et al. (15):


$$F_{\mathrm{ROH}}=\frac{\sum L_{\mathrm{ROH}}}{L_{\text{auto}}}$$

where $L_{\mathrm{ROH}}$ is the total length of all ROH in an individual, and $L_{\text{auto}}$ refers to the autosomal genome length covered by SNPs included in the array. F_HOM_ was assessed based on the proportion of homozygotes. Pearson correlation coefficients and their corresponding significance test values were calculated using the cor.test function in the R (V4.4.1) (16). To calculate correlation coefficients, F_ROH_ coefficients were also estimated for four length categories of ROH (F_ROH(1–5 Mb)_, F_ROH(5–10 Mb)_, F_ROH(10–20 Mb)_, and F_ROH(>20 Mb)_).

### 2.6 Detection of ROH islands and gene annotation

To identify the genomic regions most frequently associated with ROH, we counted the number of times SNPs were detected in ROH across all individuals to calculate the percentage of SNPs present in ROH. In this study, the top 1% of SNPs observed in ROH was chosen as the threshold for identifying the genomic region most frequently associated with ROH. A series of neighboring SNPs above this threshold formed a genomic region, which we called an ROH island.

To annotate the genes within the ROH islands, we mapped their genomic coordinates to the goat reference genome (ARS1.2, GCF_001704415.2) using NCBI’s genomic data viewer.^2^ Subsequently, all candidate genes located in these islands were subjected to functional enrichment analysis using the DAVID tool (V6.8) (17) to identify significant Gene Ontology (GO) terms and Kyoto Encyclopedia of Genes and Genomes (KEGG) pathways. Additionally, to further dissect the functional associations among candidate genes, a protein–protein interaction (PPI) network was constructed using the STRING database (v12.0) (18). Interactions with a combined score ≥ 0.4 (medium confidence) were retained, and hub genes were identified based on node degree centrality.

## 3 Results

### 3.1 Distribution of runs of homozygosity

**Figure 1:**
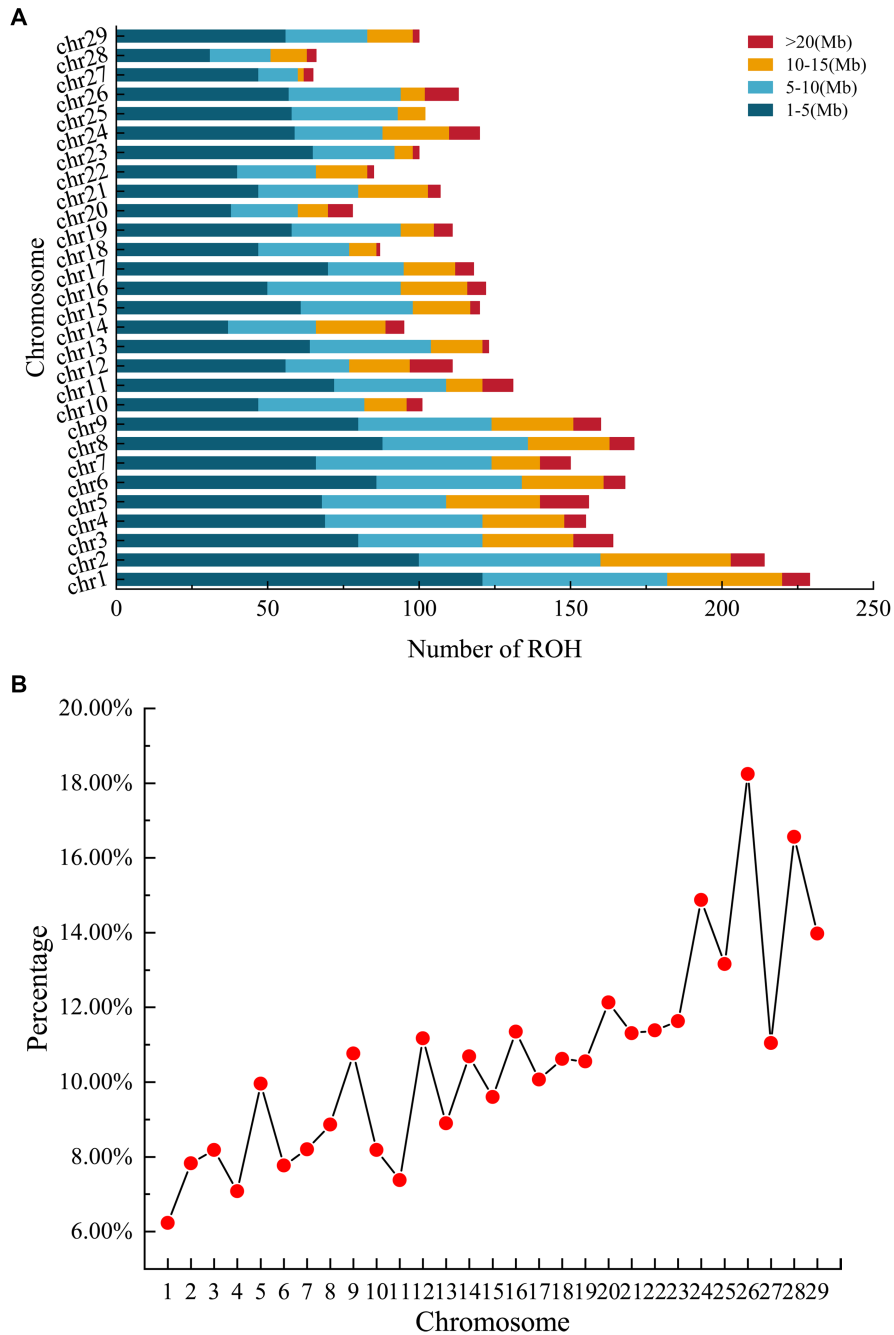
ROH patterns in Inner Mongolia cashmere goats. **(A)** The count of ROH segments exceeding 1 Mb in length per chromosome and distribution of different ROH classes per chromosome (stacked bars). **(B)** Average percentage of each chromosome covered by ROH (red dots). ROH, runs of homozygosity.

A total of 3,622 ROH segments were identified from the 380 individuals. On average, each individual carried 9.53 ROHs (range: 1–37), and the mean cumulative ROH length per individual was 71.48 Mb. All individuals possessed at least one ROH segment ≥1 Mb in length. Significant differences were observed in ROH number and coverage rate among chromosomes: chromosome 1 harbored the highest number of ROH segments (229 segments), yet exhibited the lowest coverage rate (6.23%); in contrast, chromosome 26 showed the highest coverage rate (18.24%), while chromosome 27 contained the fewest ROH segments (65 segments) (Figure 1). The longest ROH was located on chromosome 29 (28.24 Mb), and the shortest on chromosome 1 (1.95 Mb).

Regarding length distribution (Table 1), short ROHs (1–5 Mb) predominated in number (50.19%), but their cumulative genomic proportion (22.35%) was lower than that of the 5–10 Mb (27.44%) and 10–20 Mb (28.21%) categories. Notably, although ROHs longer than 20 Mb accounted for only 5.36% of the total number, their cumulative length proportion reached 22.01%, which was comparable to that of the short ROH category.

**Table 1:** Descriptive statistics of four classes of ROH in IMCG.

Class	*N*	Percentage of number, %	Mean ± SD, Mb	Total length, Mb	Percentage of length, %
ROH_1–5 Mb_	1,818	50.19%	3.34 ± 0.84	6070.26	22.35%
ROH_5–10 Mb_	1,056	29.16%	7.06 ± 1.41	7452.72	27.44%
ROH_10–20 Mb_	554	15.30%	12.83 ± 2.70	7663.09	28.21%
ROH_>20 Mb_	194	5.36%	30.82 ± 11.98	5978.23	22.01%
ROH_All_	3,622	100.00%	7.50 ± 7.33	27164.29	100.00%

### 3.2 Genomic inbreeding

**Figure 2:**
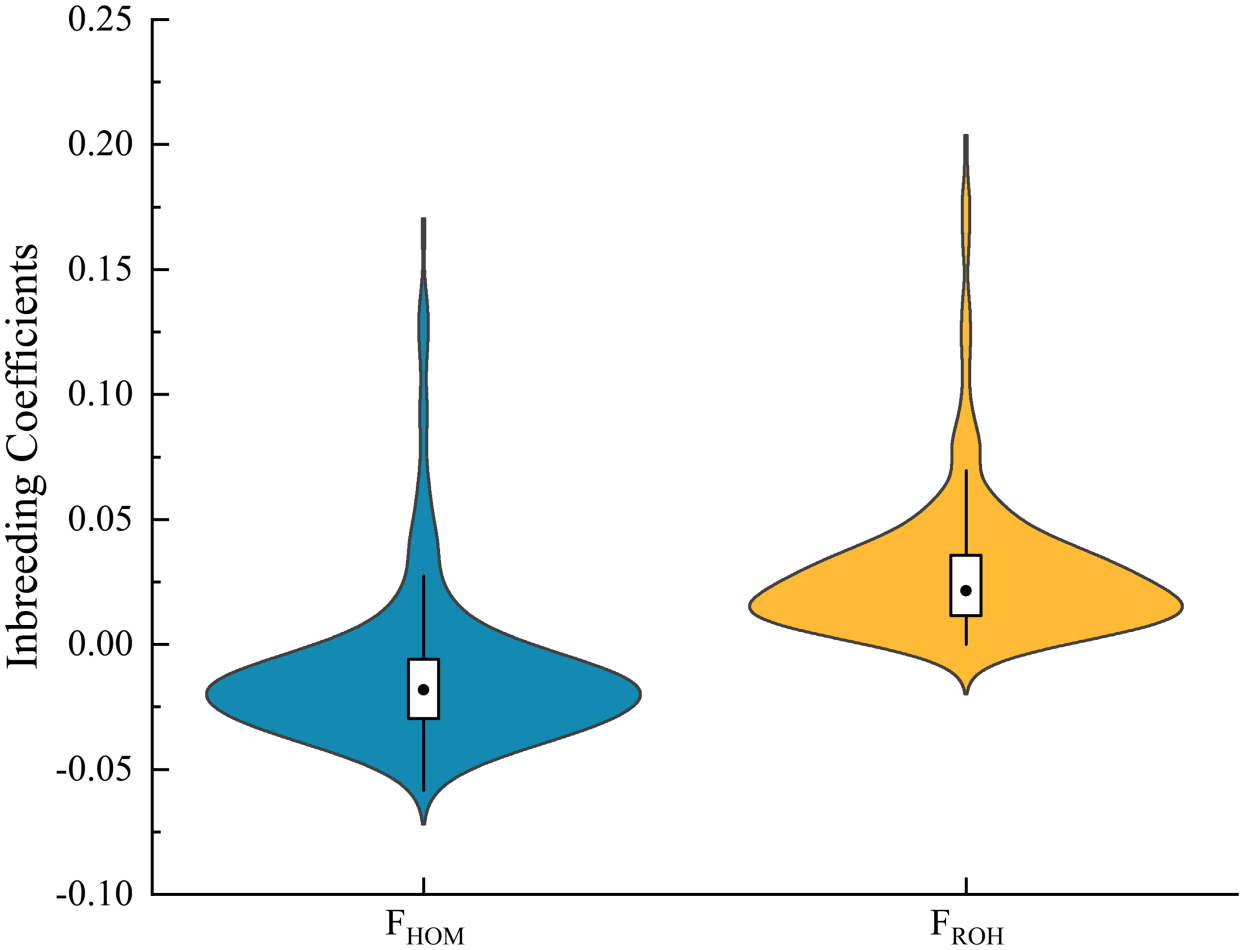
Distribution of inbreeding coefficients based on observed homozygosity (F_HOM_) and runs of homozygosity (F_ROH_) for Inner Mongolia cashmere goats.

Based on genomic data, two inbreeding coefficients were calculated, and the results are presented in Table 2 and Figure 2. The F_HOM_ ranged from −0.0583 to 0.1654 (mean ± SD: −0.0126 ± 0.0301), with most values clustering between −0.05 and 0.025. In contrast, F_ROH_ ranged from 0.0009 to 0.1991 (mean ± SD: 0.0290 ± 0.0289) and were mainly distributed between 0 and 0.05. The mean F_ROH_ values calculated for ROH length categories increased with segment length: 0.0066 ± 0.0037 (1–5 Mb), 0.0091 ± 0.0067 (5–10 Mb), 0.0129 ± 0.0094 (10–20 Mb), and 0.0228 ± 0.0234 (>20 Mb).

**Table 2:** Descriptive statistics of inbreeding coefficients for different classes in IMCG.

Inbreeding coefficients	Mean	SD	Range
F_HOM_	−0.0126	0.0301	−0.0583 to 0.1654
F_ROH_	0.0290	0.0289	0.0009–0.1991
F_ROH (1–5)_	0.0066	0.0037	0.0007–0.0208
F_ROH (5–10)_	0.0091	0.0067	0.0020–0.0459
F_ROH (10–20)_	0.0129	0.0094	0.0041–0.0633
F_ROH (>20)_	0.0228	0.0234	0.0081–0.1207

**Figure 3:**
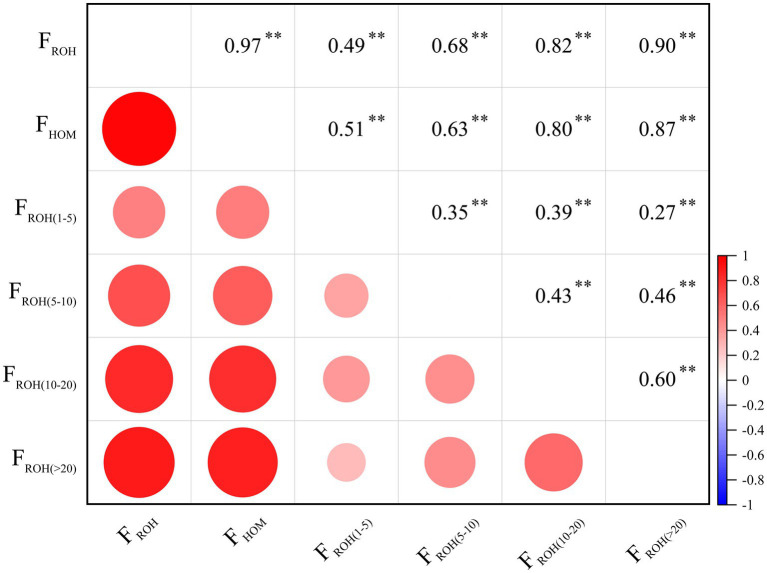
The pearson correlation between the genomic inbreeding coefficients, including F_HOM_ and F_ROH_ (F_ROH_, F_ROH(1–5 Mb)_, F_ROH(5–10 Mb)_, F_ROH(10–20 Mb)_, and F_ROH(>20 Mb)_).

Pairwise correlation analysis among these inbreeding coefficients (Figure 3) revealed the strongest association between F_ROH_ and F_HOM_ (*r* = 0.97). The correlation between F_ROH_ and the category-specific F_ROH_ estimates increased with ROH length, a pattern also observed for F_HOM_. The weakest correlation was found between F_ROH (1–5)_ and F_ROH (5–10)_ (*r* = 0.35).

### 3.3 Detection of ROH islands and gene annotation

**Figure 4:**
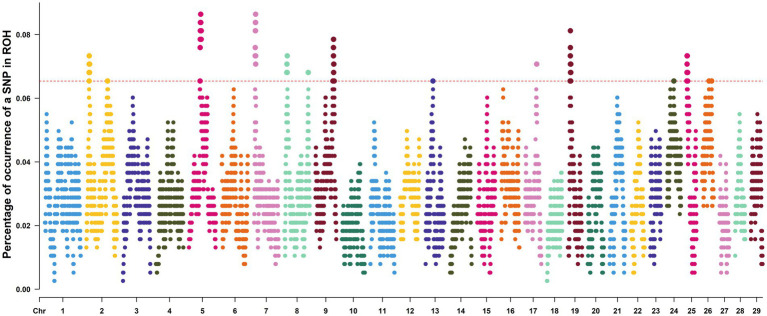
Manhattan plot of SNPs frequencies in ROH across the genome. The red line represents the ROH islands threshold for the top 1% of SNP frequency. SNPs, single nucleotide polymorphisms; ROH, runs of homozygosity.

To identify genomic regions with high-frequency homozygosity in the population, this study focused on SNPs enriched within ROH islands. Figure 4 shows the distribution of the proportion of SNPs located within ROH along the chromosomes, highlighting regions exceeding a predefined threshold. Based on this, a total of 14 ROH islands were identified across 11 chromosomes (Table 3), encompassing 589 SNPs and 238 genes (Supplementary Table S1). Among these, chromosome 25 had the highest number of annotated genes (117 in total), while no genes were annotated in specific regions of chromosomes 7, 8, 13, and 17. Many of the annotated genes are putatively associated with economically important traits, including those related to reproduction (e.g., *TXLNA*, *HDAC1*, *AQP4*) and hair follicle development (e.g., *MIR21*, *DNASE1L2*, *MMP25*).

**Table 3:** Statistics of ROH islands observed in the IMCG population.

Chromosome	Start, bp	End, bp	Length, bp	No. SNPs	No. genes
2	12,283,827	14,789,346	2,505,520	59	37
2	93,379,497	93,401,647	22,151	2	0
5	49,134,334	54,355,955	5,221,622	121	9
7	5,267,872	7,767,750	2,499,879	75	0
8	11,246,682	13,775,887	2,529,206	47	4
8	105,880,550	105,965,786	85,237	2	0
9	76,818,267	82,654,052	5,835,786	102	27
13	32,488,843	32,550,624	61,782	2	0
17	51,205,345	51,581,703	376,359	10	0
19	10,321,740	13,555,481	3,233,742	63	28
24	30,144,518	30,521,754	377,237	6	1
25	702,894	4,138,138	3,435,245	62	117
26	30,200,702	30,978,147	777,446	31	14
26	41,866,152	42,063,807	197,656	7	1

**Figure 5:**
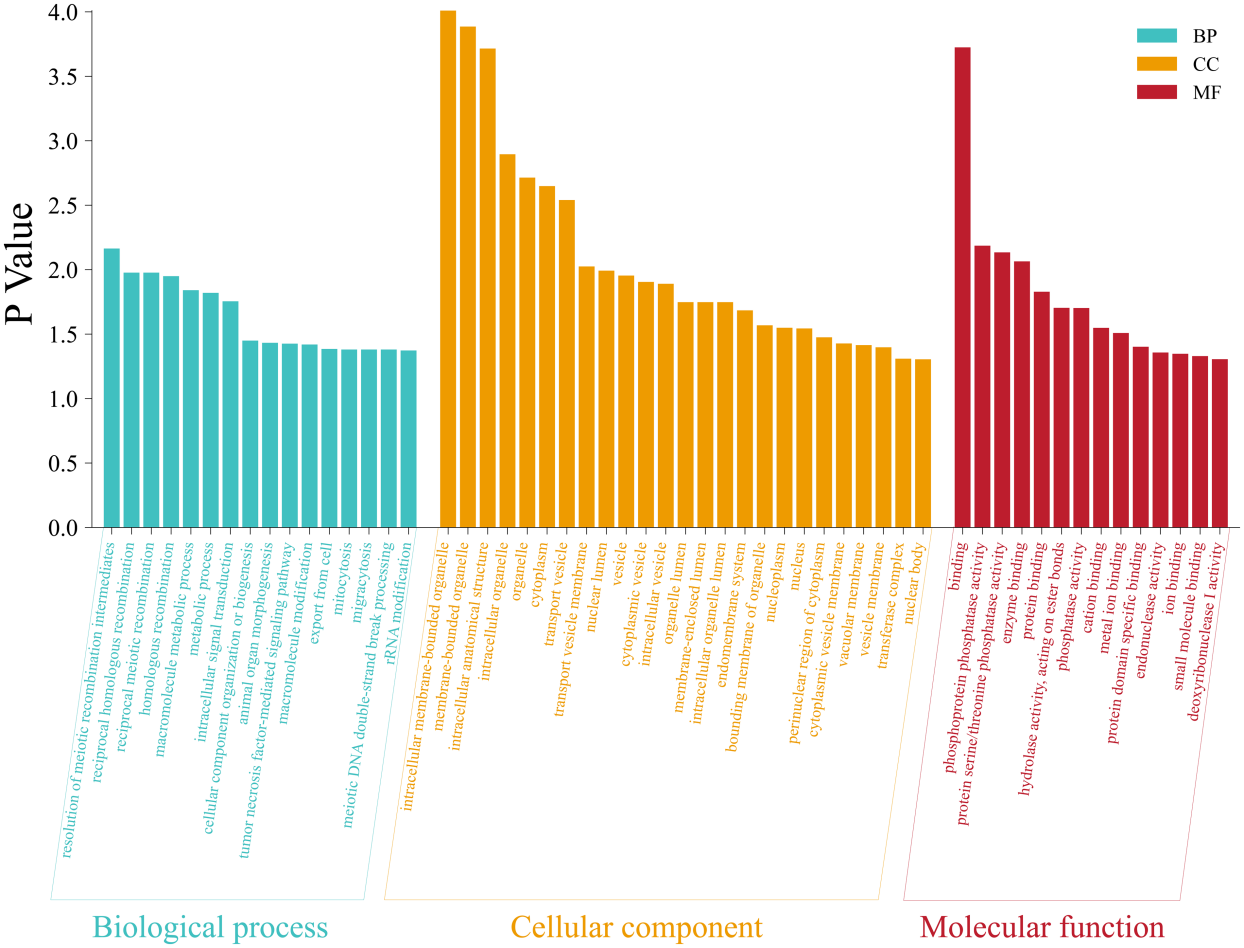
GO terms enrichment analysis of genes located within ROH islands in the Inner Mongolia cashmere goats.

**Figure 6:**
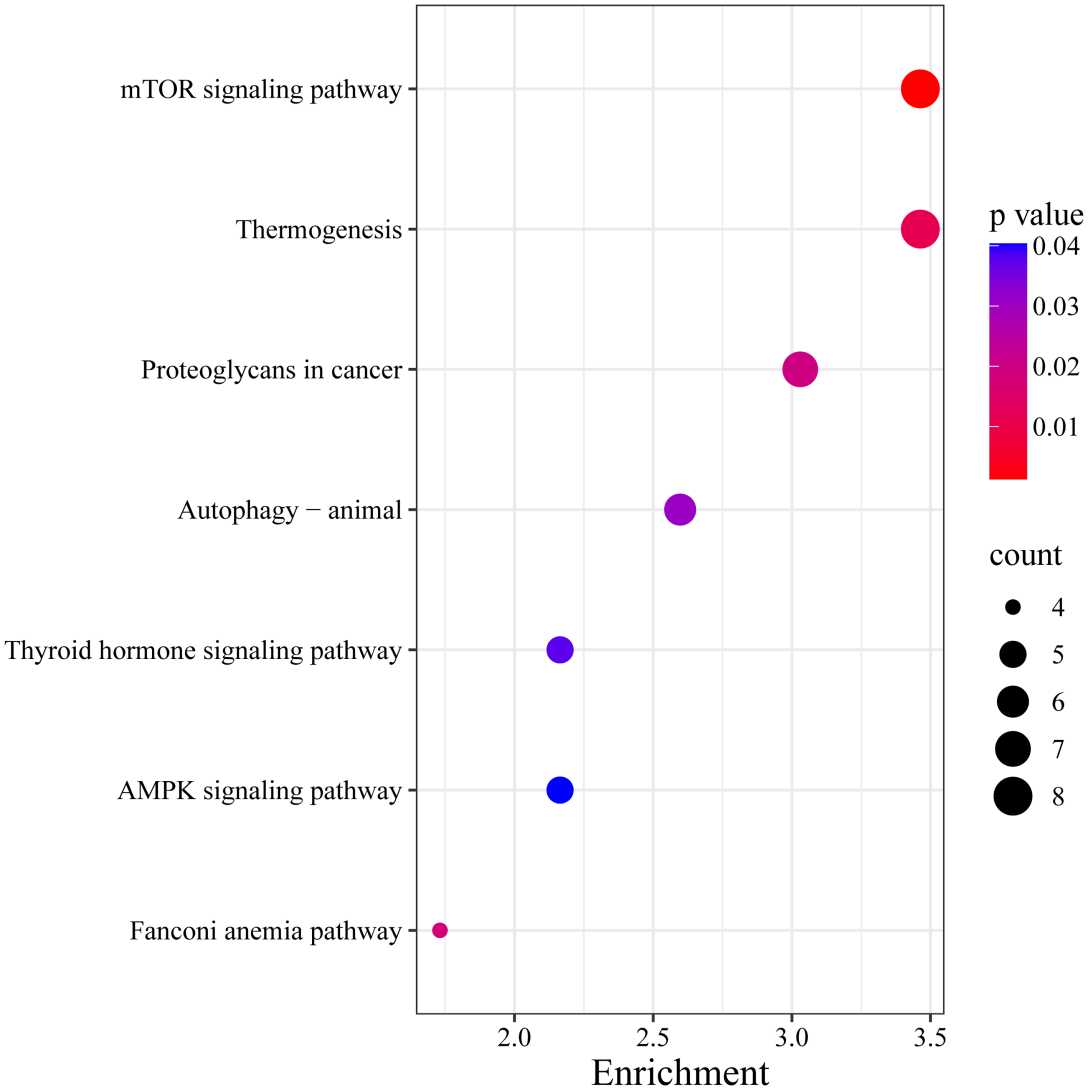
KEGG pathways enrichment analysis of genes located within ROH islands in the Inner Mongolia cashmere goats.

**Figure 7:**
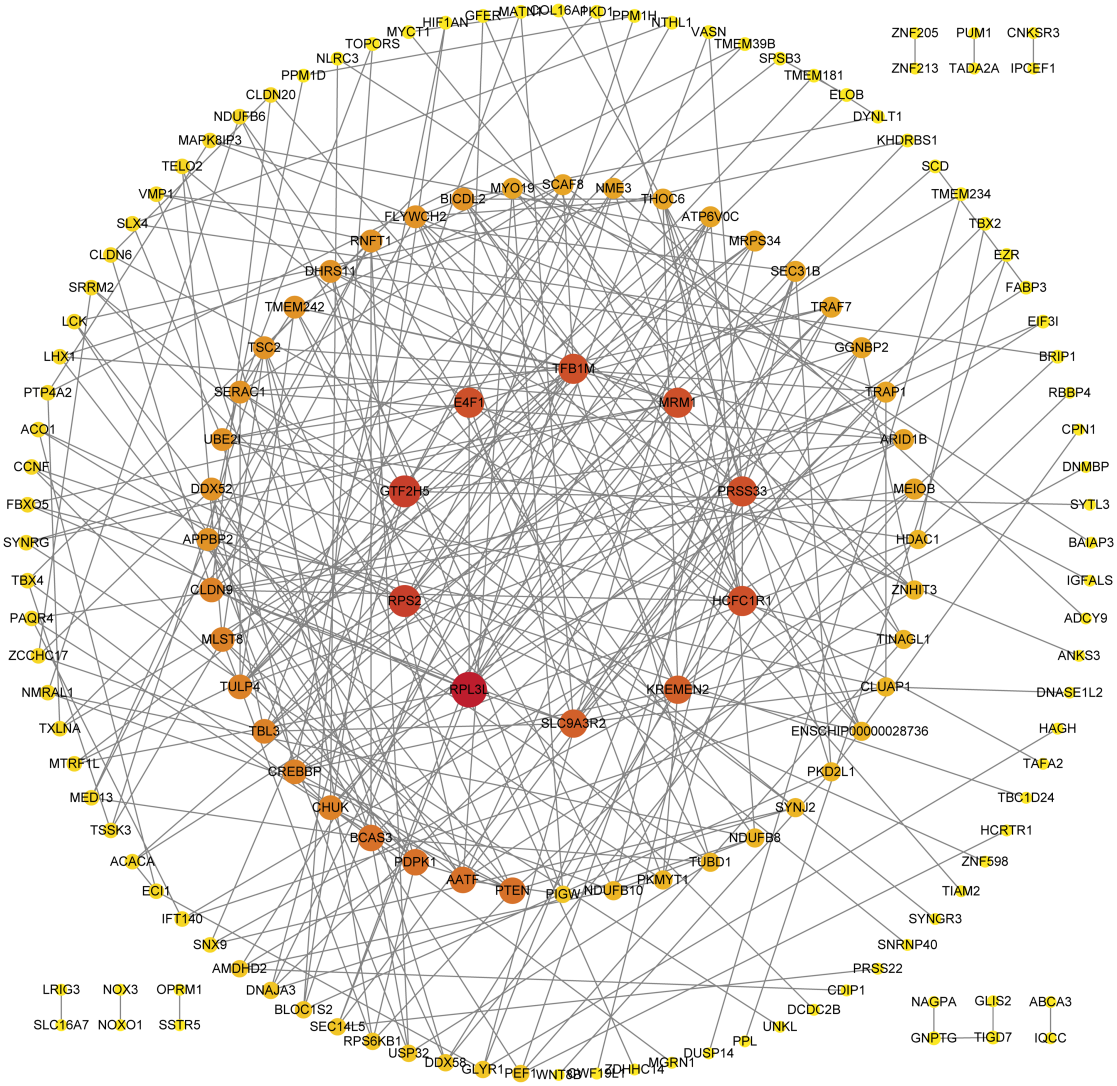
Protein–protein interaction (PPI) network of candidate genes located within ROH islands in Inner Mongolia cashmere goats.

Further functional enrichment analysis of the genes located within the ROH islands revealed significant results (Figures 5, 6): a total of 16 GO terms related to biological processes, 14 related to molecular functions, and 25 related to cellular components, along with 7 KEGG pathways, were significantly enriched (*p* < 0.05). Detailed information on the corresponding genes and functional terms is available in Supplementary Table S2. In addition, PPI network analysis revealed that these annotated genes were clustered into nine networks (Figure 7). The largest PPI network contained 142 genes, among which several genes (e.g., *RPL3L*, *RPS2*, *PTEN*, *TBL3*, *GTF2H5*, and *MRM1*) exhibited the highest node degrees.

## 4 Discussion

This study focused on the IMCG (Erlangshan subtype) due to its well-defined breeding history and accessibility. We acknowledge that the three IMCG subtypes (Albas, Erlangshan, Alashan) may exhibit some genetic differentiation due to geographical separation and breeding objectives (1). Therefore, the ROH patterns and inbreeding coefficients reported here are most directly representative of the IMCG (Erlangshan subtype) population from this specific farm. Future studies encompassing all three subtypes from multiple farms are warranted to fully capture the genomic diversity of the broader IMCG genetic resource.

### 4.1 Distribution characteristics of runs of homozygosity

A primary consideration in current ROH research is the lack of unified standards (6). The number and length of ROH identified in genotyping data largely depend on the specific parameters and thresholds applied during sequence analysis (19). Additionally, filtering SNPs prior to analysis (e.g., excluding loci with low MAF, deviation from HWE, or high LD) also significantly influences the result (20, 21). It should be noted that adopting stricter criteria, such as increasing the minimum length or reducing the number of allowed heterozygous sites, will reduce both the number of detected ROH and their total length, thereby lowering the F_ROH_ estimate. This effect is particularly pronounced in short ROH categories, which reflect ancient inbreeding events (13). Conversely, overly permissive parameters may increase the detection rate of false positive ROH (22). We fully recognize that parameter selection directly influences ROH detection outcomes and, consequently, F_ROH_ estimation. The parameter combination employed in this study represents a widely adopted balanced strategy in livestock genomics research, which was determined after careful consideration in the context of medium-density goat SNP70K BeadChip data.

Using this approach, the mean number of ROH per individual (MN_ROH_) detected in Inner Mongolia cashmere goats was 9.35, which closely aligns with the value reported for Chinese Merino (~9.23) (23), yet differs from earlier findings on the same breed by Wang et al. (1). This discrepancy highlights the influence of factors such as SNP density and analytical parameters on ROH detection outcomes (24). Analysis of ROH lengths revealed that fragments between 1 and 5 Mb constituted approximately half (50.19%) of the total, indicating the population has not recently undergone severe inbreeding. This finding aligns with reports by Zhao et al. (25). Notably, ROH longer than 20 Mb still constituted 5.36% of the total. Given that long ROH are associated with an increased risk of deleterious homozygous variants (26, 27) and can lead to harms related to inbreeding such as loss of genetic diversity and deterioration of production traits (28–31), individuals carrying such segments warrant attention due to their potential inbreeding risk.

### 4.2 Estimation and comparison of inbreeding coefficients

Traditionally, inbreeding coefficients at both the individual and population levels have been primarily calculated from pedigree data (F_PED_) using the path coefficient method proposed by Wright (32). This method relies on two key assumptions: pedigree records must be complete and accurate, and individuals within the base population must be unrelated. In practice, however, these conditions are often difficult to satisfy, and pedigree errors due to misunderstandings, misidentification, or recording inaccuracies are relatively common (33). Studies have confirmed that incomplete pedigree information or registration errors can negatively affect the genetic progress of livestock populations and the effectiveness of conservation strategies (34–36).

With the widespread adoption of high-throughput genotyping technologies, the availability of extensive genome-wide SNP data has provided new research tools for various livestock species, including goats. Utilizing this genomic information enables a more precise estimation of inbreeding coefficients, thereby enhancing our understanding of inbreeding. Unfortunately, due to the unavailability of complete pedigree data in this study, we calculated and compared F_ROH_ and F_HOM_ in IMCGs. Notably, F_ROH_ values were generally higher than F_HOM_ values, and the mean F_HOM_ was negative (−0.0126). This discrepancy can be attributed to F_HOM_ is calculated based on the observed proportion of homozygous genotypes across all loci, which cannot distinguish between homozygosity due to identity by descent (IBD) and identity by state (IBS). Consequently, F_HOM_ can be biased downward, even yielding negative values, when there is an excess of heterozygosity in the population relative to Hardy–Weinberg expectations—a phenomenon that can arise from factors such as balancing selection or population substructure (37, 38). In contrast, F_ROH_ specifically captures long, continuous homozygous segments that are highly indicative of recent autozygosity (IBD). Therefore, F_ROH_ is considered a more accurate and direct measure of genomic inbreeding for population management (3). Our observation of higher F_ROH_ relative to F_HOM_ is consistent with findings in Arbas Cashmere goats and Hu sheep studies (37, 39).

Furthermore, we analyzed the correlations between inbreeding coefficients based on ROH of different lengths with F_ROH_ and F_HOM_. The results showed a strong correlation between F_ROH_ and F_HOM_ (*r* = 0.97), which is consistent with the findings of Islam et al. (39). Similarly strong correlations have also been observed in other livestock species, such as local cattle (40), buffalo (41), and pigs (42, 43). In this study, the negative values observed for F_HOM_ may be attributed to heterozygote advantage at certain loci within the population, which increases heterozygote frequency and leads to a lower observed homozygosity than expected (37). In contrast, F_ROH_ is based on continuous homozygous segments in the genome, and its estimates are always positive. Therefore, F_ROH_ is considered a more efficient and accurate method for quantifying the level of inbreeding (3, 44).

### 4.3 Candidate genes within ROH islands and their potential functions

ROH islands refer to continuous homozygous regions in the genome that occur at high frequencies within a population (45). The formation of such regions may result from natural or artificial selection acting on specific genotypes, or from population historical events (e.g., bottleneck effects, genetic drift) or structured breeding practices (3, 22). Therefore, while ROH islands are often interpreted as potential signatures of selection, they are not definitive proof, as identical patterns can arise from neutral processes. Analyzing ROH islands aids in identifying genomic segments that have undergone natural or artificial selection and serves as an important preliminary approach for detecting candidate genes associated with complex traits (46). Consequently, this analytical method has been widely applied across various species to identify genes related to domestication or to animal production and reproductive traits.

Among the 14 ROH islands identified in this study, no annotated genes were detected in 4 islands, which may be attributed to incomplete annotation of the reference genome (47). In the remaining 10 islands, we identified a large number of candidate genes associated with various production traits in IMCGs (Supplementary Table S1). Among them, several genes are considered strong candidates for influencing reproductive processes based on prior functional studies: *PUM1* has been shown to regulate the differentiation of embryonic stem cells into oocytes in a mouse model (48); *PAQR4*, as a member of the *PAQR* family, may be involved in the progesterone secretion pathway according to a study in dairy goats (49); *ABCC2* was identified as an important regulatory factor in the transition from primordial to primary follicles in goats, with its expression declining as follicular development progresses (50); previous studies in other species suggest that *HDAC1* and *HDAC3* can cooperate with the transcription factor *YY1* to regulate *PHLDA2*, a gene implicated in placental development (51). This highlights *HDAC1* as a putative candidate influencing reproductive traits in goats, although direct functional validation in IMCG is needed. Additionally, *NDUFB6* and *NDUFB10* may contribute to maintaining sperm viability and metabolism (52), while *AQP4* and *TXLNA* could be key molecules affecting reproductive efficiency in goats (53, 54). Notably, *MIR21* is not only involved in spermatogenesis and follicular development but also serves as a critical factor in the formation and development of hair follicles in goats (47, 55, 56).

This study also identified multiple candidate genes for hair follicle development: *DNASE1L2* has been shown to play an important role in hair follicle development in Changthangi goats and is involved in wool growth regulation and fiber diameter modulation in Gansu alpine fine-wool sheep (57, 58); *MMP25* has been determined as a key gene promoting cashmere growth, with its expression significantly upregulated during the active growth phase of cashmere (59). Additionally, *LCK*, *IL32*, *MEFV*, and *NLRC3* are often associated with immune regulation (60–63); *VMP1, CREBBP*, and *CHUK* are regarded as key regulators of growth and development (64–66); while milk fat synthesis and metabolism are frequently linked to genes such as *ACACA*, *ABCA3*, *AMDHD2*, *ECI1*, and *PGP* (67, 68).

In the present study, we further conducted GO term and KEGG pathway analyses for all candidate genes located within ROH islands. Among them, three GO terms are noteworthy: animal organ morphogenesis (GO:0009887), macromolecule metabolic process (GO:0008152), and metabolic process (GO:0043170). Furthermore, accumulating evidence has implicated the *mTOR* signaling pathway in maintaining hair follicle cycling and follicular stem cell homeostasis (69). These findings collectively suggest that most quantitative phenotypic traits are likely regulated through polygenic interactions. PPI network analysis further highlighted hub genes such as *RPL3L*, *RPS2*, *PTEN*, and *TBL3* as central nodes within the interaction network (Figure 7), underscoring their potential regulatory roles in traits like hair follicle development and energy metabolism. Notably, *RPS2* participates in a conserved regulatory network responsible for 40S ribosomal subunit assembly (70). *RPL3L* is a striated muscle-specific ribosomal protein (71), and *TBL3* is a confirmed component of the *SSU* processome essential for small ribosomal subunit biogenesis (72, 73). Furthermore, *PTEN* is a negative regulator of the *mTOR* signaling pathway, and its role in hair follicle development has been demonstrated (74). The results provide novel insights into phenotypic trait selection and genetic architecture underlying complex traits. However, it should be noted that the functional information derived from GO and KEGG analyses remains limited in scope. The incomplete annotation of the goat genome and the physical clustering of genes within ROH islands may bias the functional interpretation (38, 46). Future functional validation studies, including tissue-specific expression profiling of key candidate genes (e.g., *RPS2*, *RPL3L*, *PTEN*) in relevant tissues such as skin and hair follicles, are needed to confirm their roles in cashmere production and metabolic traits.

## 5 Conclusion

In this study, we investigated the distribution of ROH across autosomes and inbreeding coefficients in IMCG (Erlangshan subtype). The ROH segments spanning 1–5 Mb exhibited both the highest frequency and the most substantial cumulative length proportion. Notably, ROH fragments exceeding 20 Mb accounted for 22.01% of the total ROH length, demonstrating a comparable contribution to genomic coverage as the 1–5 Mb category. Correlation analyses among different inbreeding estimators revealed the strongest association between F_ROH_ and F_HOM_. The IMCG population displayed relatively low inbreeding coefficients, suggesting effective management of inbreeding depression. ROH islands were found to encompass multiple candidate genes associated with crucial biological functions including reproductive regulation, hair follicle morphogenesis and development, growth traits, and milk fat metabolism. These findings provide valuable insights for implementing genomic selection strategies in IMCGs while maintaining controlled inbreeding levels. Furthermore, this research establishes a foundation for elucidating the molecular mechanisms underlying economically important traits, thereby facilitating precision breeding programs through targeted utilization of homozygosity patterns.

## Data Availability

The datasets presented in this study can be found in online repositories. The datasets generated for this study are available in the Figshare repository under DOI: https://doi.org/10.6084/m9.figshare.29224259.

## Footnotes

1
https://doi.org/10.6084/m9.figshare.29224259

2
https://www.ncbi.nlm.nih.gov/gdv/browser/genome/?id=GCF_001704415.2

## References

[1] Wang R Wang X Qi Y Li Y Na Q Yuan H . Genetic diversity analysis of Inner Mongolia cashmere goats (Erlangshan subtype) based on whole genome re-sequencing. BMC Genomics. (2024) 25:698. doi: 10.1186/s12864-024-10485-x, 39014331 PMC11253418

[2] Li X Su R Wan W Zhang W Jiang H Qiao X . Identification of selection signals by large-scale whole-genome resequencing of cashmere goats. Sci Rep. (2017) 7:15142. doi: 10.1038/s41598-017-15516-0, 29123196 PMC5680388

[3] Ceballos FC Joshi PK Clark DW Ramsay M Wilson JF. Runs of homozygosity: windows into population history and trait architecture. Nat Rev Genet. (2018) 19:220–34. doi: 10.1038/nrg.2017.109, 29335644

[4] Silva GAA Harder AM Kirksey KB Mathur S Willoughby JR. Detectability of runs of homozygosity is influenced by analysis parameters and population-specific demographic history. PLoS Comput Biol. (2024) 20:e1012566. doi: 10.1371/journal.pcbi.1012566, 39480880 PMC11556709

[5] Ino Č Maja F Johann S. Genomic dissection of inbreeding depression: a gate to new opportunities. Rev Bras Zootec. (2017) 46:773–82. doi: 10.1590/s1806-92902017000900010

[6] Peripolli E Munari DP Silva M Lima ALF Irgang R Baldi F. Runs of homozygosity: current knowledge and applications in livestock. Anim Genet. (2017) 48:255–71. doi: 10.1111/age.12526, 27910110

[7] Ma R Liu J Ma X Yang J. Genome-wide runs of homozygosity reveal inbreeding levels and trait-associated candidate genes in diverse sheep breeds. Genes. (2025) 16:316. doi: 10.3390/genes16030316, 40149467 PMC11942120

[8] Hervás-Rivero C Mejuto-Vázquez N López-Carbonell D Altarriba J Diaz C Molina A . Runs of Homozygosity Islands in autochthonous Spanish cattle breeds. Genes. (2024) 15:1477. doi: 10.3390/genes15111477, 39596677 PMC11593383

[9] Rostamzadeh Mahdabi E Esmailizadeh A Han J Wang MS. Comparative analysis of runs of Homozygosity Islands in indigenous and commercial chickens revealed candidate loci for disease resistance and production traits. Vet Med Sci. (2025) 11:e70074. doi: 10.1002/vms3.70074, 39655377 PMC11629026

[10] Moore D. Purification and concentration of DNA from aqueous solutions: preparation and analysis of DNA. Curr Protoc Mol Biol. (1994) 25:2.1.1–9. doi: 10.1002/j.1934-3647.1994.tb00220.x 34266225

[11] Purcell S Neale B Todd-Brown K Thomas L Ferreira MA Bender D . PLINK: a tool set for whole-genome association and population-based linkage analyses. Am J Hum Genet. (2007) 81:559–75. doi: 10.1086/519795, 17701901 PMC1950838

[12] Lencz T Lambert C DeRosse P Burdick KE Morgan TV Kane JM . Runs of homozygosity reveal highly penetrant recessive loci in schizophrenia. Proc Natl Acad Sci USA. (2007) 104:19942–7. doi: 10.1073/pnas.0710021104, 18077426 PMC2148402

[13] Ferenčaković M Sölkner J Curik I. Estimating autozygosity from high-throughput information: effects of SNP density and genotyping errors. GSE. (2013) 45:42. doi: 10.1186/1297-9686-45-42, 24168655 PMC4176748

[14] Mastrangelo S Tolone M Sardina MT Sottile G Sutera AM Di Gerlando R . Genome-wide scan for runs of homozygosity identifies potential candidate genes associated with local adaptation in Valle del Belice sheep. Genet Sel Evol. (2017) 49:84. doi: 10.1186/s12711-017-0360-z, 29137622 PMC5684758

[15] McQuillan R Leutenegger AL Abdel-Rahman R Franklin CS Pericic M Barac-Lauc L . Runs of homozygosity in European populations. Am J Hum Genet. (2008) 83:359–72. doi: 10.1016/j.ajhg.2008.08.007, 18760389 PMC2556426

[16] R Core Team (2024). R: A language and environment for statistical computing. Vienna, Austria: R Foundation for Statistical Computing. Available online at: https://www.R-project.org

[17] Huang da W Sherman BT Lempicki RA. Systematic and integrative analysis of large gene lists using DAVID bioinformatics resources. Nat Protoc. (2009) 4:44–57. doi: 10.1038/nprot.2008.211, 19131956

[18] Szklarczyk D Kirsch R Koutrouli M Nastou K Mehryary F Hachilif R . The STRING database in 2023: protein-protein association networks and functional enrichment analyses for any sequenced genome of interest. Nucleic Acids Res. (2023) 51:D638–d646. doi: 10.1093/nar/gkac1000, 36370105 PMC9825434

[19] Howrigan DP Simonson MA Keller MC. Detecting autozygosity through runs of homozygosity: a comparison of three autozygosity detection algorithms. BMC Genomics. (2011) 12:460. doi: 10.1186/1471-2164-12-460, 21943305 PMC3188534

[20] Albrechtsen A Nielsen FC Nielsen R. Ascertainment biases in SNP chips affect measures of population divergence. Mol Biol Evol. (2010) 27:2534–47. doi: 10.1093/molbev/msq148, 20558595 PMC3107607

[21] Wigginton JE Cutler DJ Abecasis GR. A note on exact tests of hardy-Weinberg equilibrium. Am J Hum Genet. (2005) 76:887–93. doi: 10.1086/429864, 15789306 PMC1199378

[22] Meyermans R Gorssen W Buys N Janssens S. How to study runs of homozygosity using PLINK? A guide for analyzing medium density SNP data in livestock and pet species. BMC Genomics. (2020) 21:94. doi: 10.1186/s12864-020-6463-x, 31996125 PMC6990544

[23] He S Di J Han B Chen L Liu M Li W. Genome-wide scan for runs of homozygosity identifies candidate genes related to economically important traits in Chinese merino. Animals. (2020) 10:524. doi: 10.3390/ani10030524, 32245132 PMC7143548

[24] Macciotta NPP Colli L Cesarani A Ajmone-Marsan P Low WY Tearle R . The distribution of runs of homozygosity in the genome of river and swamp buffaloes reveals a history of adaptation, migration and crossbred events. GSE. (2021) 53:20. doi: 10.1186/s12711-021-00616-3, 33639853 PMC7912491

[25] Zhao Q Huang C Chen Q Su Y Zhang Y Wang R . Genomic inbreeding and runs of homozygosity analysis of cashmere goat. Animals. (2024) 14:1246. doi: 10.3390/ani14081246, 38672394 PMC11047310

[26] Pemberton TJ Szpiech ZA. Relationship between deleterious variation, genomic autozygosity, and disease risk: insights from the 1000 genomes project. Am J Hum Genet. (2018) 102:658–75. doi: 10.1016/j.ajhg.2018.02.013, 29551419 PMC5985279

[27] Szpiech ZA Xu J Pemberton TJ Peng W Zöllner S Rosenberg NA . Long runs of homozygosity are enriched for deleterious variation. Am J Hum Genet. (2013) 93:90–102. doi: 10.1016/j.ajhg.2013.05.003, 23746547 PMC3710769

[28] Bjelland DW Weigel KA Vukasinovic N Nkrumah JD. Evaluation of inbreeding depression in Holstein cattle using whole-genome SNP markers and alternative measures of genomic inbreeding. J Dairy Sci. (2013) 96:4697–706. doi: 10.3168/jds.2012-6435, 23684028

[29] González-Recio O López de Maturana E Gutiérrez JP. Inbreeding depression on female fertility and calving ease in Spanish dairy cattle. J Dairy Sci. (2007) 90:5744–52. doi: 10.3168/jds.2007-0203, 18024768

[30] Mc Parland S Kearney JF Rath M Berry DP. Inbreeding effects on milk production, calving performance, fertility, and conformation in Irish Holstein-Friesians. J Dairy Sci. (2007) 90:4411–9. doi: 10.3168/jds.2007-0227, 17699061

[31] Miglior F Burnside EB Kennedy BW. Production traits of Holstein cattle: estimation of nonadditive genetic variance components and inbreeding depression. J Dairy Sci. (1995) 78:1174–80. doi: 10.3168/jds.S0022-0302(95)76735-2, 7622728

[32] Wright S. Coefficients of inbreeding and relationship. Am Nat. (1922) 56:330–8. doi: 10.1086/279872

[33] Ron M Blanc Y Band M Ezra E Weller JI. Misidentification rate in the Israeli dairy cattle population and its implications for genetic improvement. J Dairy Sci. (1996) 79:676–81. doi: 10.3168/jds.S0022-0302(96)76413-5, 8744233

[34] Banos G Wiggans GR Powell RL. Impact of paternity errors in cow identification on genetic evaluations and international comparisons. J Dairy Sci. (2001) 84:2523–9. doi: 10.3168/jds.S0022-0302(01)74703-0, 11768094

[35] Oliehoek PA Bijma P. Effects of pedigree errors on the efficiency of conservation decisions. GSE. (2009) 41:9. doi: 10.1186/1297-9686-41-9, 19284686 PMC3225833

[36] Sanders K Bennewitz J Kalm E. Wrong and missing sire information affects genetic gain in the Angeln dairy cattle population. J Dairy Sci. (2006) 89:315–21. doi: 10.3168/jds.S0022-0302(06)72096-3, 16357295

[37] Bao J Xiong J Huang J Yang P Shang M Zhang L. Genetic diversity, selection signatures, and genome-wide association study identify candidate genes related to litter size in Hu sheep. Int J Mol Sci. (2024) 25:9397. doi: 10.3390/ijms25179397, 39273345 PMC11395453

[38] Wang J. Marker-based estimates of relatedness and inbreeding coefficients: an assessment of current methods. J Evol Biol. (2014) 27:518–30. doi: 10.1111/jeb.12315 24444019

[39] Islam R Liu Z Li Y Jiang L Ma Y. Conservation assessment of the state goat farms by using SNP genotyping data. Genes. (2020) 11:652. doi: 10.3390/genes11060652, 32545749 PMC7349881

[40] Xu L Zhao G Yang L Zhu B Chen Y Zhang L . Genomic patterns of homozygosity in Chinese local cattle. Sci Rep. (2019) 9:16977. doi: 10.1038/s41598-019-53274-3, 31740716 PMC6861314

[41] Liu SH Ma XY Hassan FU Gao TY Deng TX. Genome-wide analysis of runs of homozygosity in Italian Mediterranean buffalo. J Dairy Sci. (2022) 105:4324–34. doi: 10.3168/jds.2021-21543, 35307184

[42] Jiang Y Li X Liu J Zhang W Zhou M Wang J . Genome-wide detection of genetic structure and runs of homozygosity analysis in Anhui indigenous and Western commercial pig breeds using PorcineSNP80k data. BMC Genomics. (2022) 23:373. doi: 10.1186/s12864-022-08583-9, 35581549 PMC9115978

[43] Shi L Wang L Liu J Deng T Yan H Zhang L . Estimation of inbreeding and identification of regions under heavy selection based on runs of homozygosity in a large white pig population. J Anim Sci Biotechnol. (2020) 11:46. doi: 10.1186/s40104-020-00447-0, 32355558 PMC7187514

[44] Dadousis C Ablondi M Cipolat-Gotet C van Kaam JT Finocchiaro R Marusi M . Genomic inbreeding coefficients using imputation genotypes: assessing the effect of ancestral genotyping in Holstein-Friesian dairy cows. J Dairy Sci. (2024) 107:5869–80. doi: 10.3168/jds.2024-24042, 38490541

[45] Di Gregorio P Perna A Di Trana A Rando A. Identification of ROH Islands conserved through generations in pigs belonging to the Nero Lucano breed. Genes. (2023) 14:1503. doi: 10.3390/genes14071503, 37510406 PMC10378754

[46] Gorssen W Meyermans R Janssens S Buys N. A publicly available repository of ROH islands reveals signatures of selection in different livestock and pet species. GSE. (2021) 53:2. doi: 10.1186/s12711-020-00599-7, 33397285 PMC7784028

[47] Xiang B Li Y Li J Zhang B Li J Jiang H . MiR-21 regulated hair follicle cycle development in cashmere goats by targeting FGF18 and SMAD7. Anim Biotechnol. (2023) 34:4695–702. doi: 10.1080/10495398.2023.2186891, 36897050

[48] Malik HN Singhal DK Saini S Malakar D. Derivation of oocyte-like cells from putative embryonic stem cells and parthenogenetically activated into blastocysts in goat. Sci Rep. (2020) 10:10086. doi: 10.1038/s41598-020-66609-2, 32572061 PMC7308273

[49] Sun S Li C Liu S Luo J Chen Z Zhang C . RNA sequencing and differential expression reveals the effects of serial oestrus synchronisation on ovarian genes in dairy goats. Reprod Fertil Dev. (2018) 30:1622–33. doi: 10.1071/rd17511, 29875030

[50] Guerreiro DD de Lima LF Mbemya GT Maside CM Miranda AM Tavares KCS . ATP-binding cassette (ABC) transporters in caprine preantral follicles: gene and protein expression. Cell Tissue Res. (2018) 372:611–20. doi: 10.1007/s00441-018-2804-3, 29488001

[51] Wang R Su L Yu S Ma X Jiang C Yu Y. Inhibition of PHLDA2 transcription by DNA methylation and YY1 in goat placenta. Gene. (2020) 739:144512. doi: 10.1016/j.gene.2020.144512, 32112983

[52] Li C Liang J Allai L Badaoui B Shao Q Ouyang Y . Integrating proteomics and metabolomics to evaluate impact of semen collection techniques on the quality and cryotolerance of goat semen. Sci Rep. (2024) 14:29489. doi: 10.1038/s41598-024-80556-2, 39604559 PMC11603158

[53] Arrighi S Bosi G Frattini S Coizet B Groppetti D Pecile A. Morphology and aquaporin immunohistochemistry of the uterine tube of Saanen goats (*Capra hircus*): comparison throughout the reproductive cycle. Reprod Domest Anim. (2016) 51:360–9. doi: 10.1111/rda.12687, 27020623

[54] Liu Y Zhou Z He X Tao L Jiang Y Lan R . Integrated analyses of miRNA-mRNA expression profiles of ovaries reveal the crucial interaction networks that regulate the prolificacy of goats in the follicular phase. BMC Genomics. (2021) 22:812. doi: 10.1186/s12864-021-08156-2, 34763659 PMC8582148

[55] Wu J Liao M Zhu H Kang K Mu H Song W . CD49f-positive testicular cells in Saanen dairy goat were identified as spermatogonia-like cells by miRNA profiling analysis. J Cell Biochem. (2014) 115:1712–23. doi: 10.1002/jcb.24835, 24817091

[56] Zi XD Lu JY Ma L. Identification and comparative analysis of the ovarian microRNAs of prolific and non-prolific goats during the follicular phase using high-throughput sequencing. Sci Rep. (2017) 7:1921. doi: 10.1038/s41598-017-02225-x, 28507337 PMC5432505

[57] Ahlawat S Arora R Sharma R Sharma U Kaur M Kumar A . Skin transcriptome profiling of Changthangi goats highlights the relevance of genes involved in pashmina production. Sci Rep. (2020) 10:6050. doi: 10.1038/s41598-020-63023-6, 32269277 PMC7142143

[58] He Z Zhao F Sun H Hu J Wang J Liu X . Screened of long non-coding RNA related to wool development and fineness in Gansu alpine fine-wool sheep. BMC Genomics. (2025) 26:8. doi: 10.1186/s12864-024-11195-0, 39762742 PMC11702032

[59] Bhat B Yaseen M Singh A Ahmad SM Ganai NA. Identification of potential key genes and pathways associated with the pashmina fiber initiation using RNA-Seq and integrated bioinformatics analysis. Sci Rep. (2021) 11:1766. doi: 10.1038/s41598-021-81471-6, 33469142 PMC7815713

[60] Asif AR Qadri S Ijaz N Javed R Ansari AR Awais M . Genetic signature of strong recent positive selection at interleukin-32 gene in goat. Asian Australas J Anim Sci. (2017) 30:912–9. doi: 10.5713/ajas.15.0941, 27165029 PMC5495668

[61] Omar AI Alam MBB Notter DR Zhao S Faruque MO Thi TNT . Association of single nucleotide polymorphism in NLRC3, NLRC5, HIP1, and LRP8 genes with fecal egg counts in goats naturally infected with Haemonchus contortus. Trop Anim Health Prod. (2020) 52:1583–98. doi: 10.1007/s11250-019-02154-z, 31828571

[62] Ross JJ. Goats, germs, and fever: are the pyrin mutations responsible for familial Mediterranean fever protective against brucellosis? Med Hypotheses. (2007) 68:499–501. doi: 10.1016/j.mehy.2006.07.027, 17005326

[63] Wang A Chao T Ji Z Xuan R Liu S Guo M . Transcriptome analysis reveals potential immune function-related regulatory genes/pathways of female Lubo goat submandibular glands at different developmental stages. PeerJ. (2020) 8:e9947. doi: 10.7717/peerj.9947, 33083113 PMC7547598

[64] Chang L Zheng Y Li S Niu X Huang S Long Q . Identification of genomic characteristics and selective signals in Guizhou black goat. BMC Genomics. (2024) 25:164. doi: 10.1186/s12864-023-09954-6, 38336605 PMC10854126

[65] Fu J Liu J Zou X Deng M Liu G Sun B . Author correction: transcriptome analysis of mRNA and miRNA in the development of LeiZhou goat muscles. Sci Rep. (2024) 14:17136. doi: 10.1038/s41598-024-67345-7, 39060310 PMC11282204

[66] Zhou X Yan Q Yang H Ren A He Z Tan Z. Maternal intake restriction programs the energy metabolism, clock circadian regulator and mTOR signals in the skeletal muscles of goat offspring probably via the protein kinase A-cAMP-responsive element-binding proteins pathway. Anim Nutr. (2021) 7:1303–14. doi: 10.1016/j.aninu.2021.09.006, 34786503 PMC8567324

[67] Selionova M Trukhachev V Aibazov M Sermyagin A Belous A Gladkikh M . Genome-wide association study of Milk composition in Karachai goats. Animals. (2024) 14:327. doi: 10.3390/ani14020327, 38275787 PMC10812594

[68] Tian P Luo Y Li X Tian J Tao S Hua C . Negative effects of long-term feeding of high-grain diets to lactating goats on milk fat production and composition by regulating gene expression and DNA methylation in the mammary gland. J Anim Sci Biotechnol. (2017) 8:74. doi: 10.1186/s40104-017-0204-2, 29026537 PMC5623059

[69] Tu W Cao YW Sun M Liu Q Zhao HG. mTOR signaling in hair follicle and hair diseases: recent progress. Front Med. (2023) 10:1209439. doi: 10.3389/fmed.2023.1209439, 37727765 PMC10506410

[70] Landry-Voyer AM Mir Hassani Z Avino M Bachand F. Ribosomal protein uS5 and friends: protein-protein interactions involved in ribosome assembly and beyond. Biomolecules. (2023) 13:853. doi: 10.3390/biom13050853, 37238722 PMC10216425

[71] Chaillou T. Ribosome specialization and its potential role in the control of protein translation and skeletal muscle size. J Appl Physiol. (2019) 127:599–607. doi: 10.1152/japplphysiol.00946.2018, 30605395

[72] Wada K Sato M Araki N Kumeta M Hirai Y Takeyasu K . Dynamics of WD-repeat containing proteins in SSU processome components. Biochem Cell Biol. (2014) 92:191–9. doi: 10.1139/bcb-2014-0007, 24754225

[73] Li K Xie X Gao R Chen Z Yang M Wen Z . Spatiotemporal protein interactome profiling through condensation-enhanced photocrosslinking. Nat Chem. (2025) 17:111–23. doi: 10.1038/s41557-024-01663-1, 39501047

[74] Liang W Chen X Ni N Zhuang C Yu Z Xu Z . Corticotropin-releasing hormone inhibits autophagy by suppressing PTEN to promote apoptosis in dermal papilla cells. Ann Med. (2025) 57:2490823. doi: 10.1080/07853890.2025.2490823, 40219757 PMC11995766

